# Analysis of the Surface Condition and Changes in Crystallographic Structure of Zirconium Oxide Affected by Mechanical Processing

**DOI:** 10.3390/ma14144042

**Published:** 2021-07-20

**Authors:** Kinga Regulska, Bartłomiej Januszewicz, Leszek Klimek, Aleksandra Palatyńska-Ulatowska

**Affiliations:** 1 Institute of Materials Science and Engineering, Faculty of Mechanical Engineering, Lodz University of Technology, 1/15 Stefanowskiego Str., 90-924 Łódź, Poland; kinga.regulska@dokt.p.lodz.pl (K.R.); bartlomiej.januszewicz@p.lodz.pl (B.J.); leszek.klimek@p.lodz.pl (L.K.); 2 Department of Endodontics, Chair of Conservative Dentistry and Endodontics, Medical University of Lodz, 251 Pomorska Str., 92-213 Łódź, Poland

**Keywords:** zirconium oxide, XRD, phase transformation, crystallographic structure, ceramics

## Abstract

Zirconium oxide is a material commonly used in dental prosthetics for making cups of permanent prosthetic restorations. In order to properly prepare the surface of zirconium oxide for prosthetic treatment, it must be veneered with ceramics. The quality of cup-veneered ceramics is dependent on many factors, including the surface free energy (SFE) and transformation of zirconium oxide. The aim of the study was to investigate the type of phase transition and the value of free energy of the surface subjected to machining (wet and dry grinding, polishing). Quantitative and qualitative phase identification measurements showed that mechanical treatment causes transformation of the tetragonal phase into a monoclinic phase in the zirconium oxide surface. Prepared samples were analyzed by means of X-ray diffraction (XRD), which confirmed the phenomenon of transition. Measurements of the wetting angle and the calculated values of the surface free energy (SFE) showed no significant differences between the samples subjected to each treatment

## 1. Introduction

Due to growing aesthetic expectations, different methods have been sought to eliminate metal from the foundation of permanent restorations. Many years of clinical observations and research have proven that metal–ceramic restorations are characterized by adequate durability and good strength properties [1,2,3,4]. Their disadvantages are worse aesthetics and biocompatibility [5]. Good aesthetic values and biocompatibility can be found with full-ceramic restorations made of zirconium oxide [5,6,7,8,9]. ZrO_2_ is an oxide–ceramic that can be used in a patient’s oral cavity. It has many beneficial properties like a high biological tolerance and a light color. For this reason, it is one of the best materials for prosthetic reconstruction [1,2,10,11]. Thanks to the use of zirconium, it is possible to make a restoration without a metal substructure, thus avoiding corrosion or causing discoloration of tissues, which occurs in the oral cavity. The fabrication of a metal substructure can be inaccurate, due to the stages of manual modeling and casting, which may result in a mismatch in a precise prosthetic element. Significant development of CAD–CAM computer technologies has been ongoing since the 1990s; they are used in the production of zirconium oxide elements, which allow high precision restorations. While a clinically acceptable marginal seal of prosthetic reconstructions amounts to 100 μm, CAD/CAM technology allows to obtain a seal up of to 30 μm. This material is used in dentistry for implants and their connectors, superstructures of permanent restorations based on implants, orthodontic brackets, post-and-core crowns, and substructures for full-contour crowns and bridges [3,4,12,13,14]. The restorations are milled from already-prepared zirconium blocks. There are two types: fully sintered blocks, in which no shrinkage of material in the final phase of milling occurs (the restoration is difficult to process and its mechanical properties worsen) and blocks that have been initially sintered, for which the final mechanical properties are obtained after the processing. This causes a slight structural shrinkage only during treatment [2,3,9,14]. In a basic form zirconia is very light and milky white in color. It is a non-transparent material. Thanks to this, it ideally masks a prepared dental abutment. Staining techniques are frequently used prior to the final sintering of the zirconium oxide framework to achieve a tooth-like color and desired aesthetics [5,7,14]. Zirconium oxide used for prosthetic restorations consists of crystal grains of 0.2–0.5 μm, and do not contain glass additives [12]. Its advantages are also its mechanical strength and abrasion resistance. Mechanical features of zirconium oxide are close to the characteristics of stainless steel. Its tensile strength equals 900–1200 MPa, its compressive strength is 2000 MPa and it has a cracking strength of 4–6 MPa. Restorations made of this material can carry loads of 750 N [12,15,16] and it occurs in three structural phases: monoclinic, tetragonal, and the cubic one. In prosthetics a tetragonal phase is used, which does not have a glassy phase. It is most advantageous from a biomechanical standpoint, because it can be stabilized at room temperature by adding oxides of magnesium, calcium, yttrium, or cerium [1,2,10,11].

The monoclinic phase is stable at low temperatures. Heating it up to around 1200 °C leads to a transformation into a tetragonal phase and it is stable up to 2370 °C. A volume shrinkage about 8%, as well as an increase in density accompany this transformation. During the process of cooling, at about 1000 °C, a reversible transition into a monoclinic phase occurs together with a decrease in density and an increase in volume. Additionally, while cooling after the sintering, residual tensions may emerge. It influences the bonding and the strength of veneering ceramics [14,17,18]. Thus, a tetragonal phase is not stable in the room temperature. For a better resistance it is stabilized with yttrium or calcium oxide to obtain a metastable phase. Under external stimuli, e.g., stress, it can transform into a stable form at this temperature in the monoclinic phase.

Additionally, a very important feature that classifies zirconium oxide as a dental material is its self-healing ability, which is connected to the increase in volume during the transition from a tetragonal to a monoclinic phase. Compared with other oxide ceramics, it is also very resistant to crack formation. These properties result from mixing zirconium oxide with an appropriate amount of yttrium oxide, which triggers the so-called amplifying transformation, stabilizing the zirconium oxide in its tetragonal high-temperature phase. Then, by introducing external energy, e.g., in a situation where scratches are formed, individual ZrO_2_ grains transform locally. The crystals increase in volume at ambient temperature and transform into a stable monoclinic system; the so-called strengthening transformation [3]. Compressive stresses in the structure caused by the increase in volume and the energy consumed during the transformation lead to a slowing down or stopping of the propagation of scratches or cracks [6,14]. It turns out that, before the propagation of a crack, a localized stress can be enough for a transformation to start in the crack’s apex region. In this case, a 4% increase in material volume is beneficial [14]. Thanks to this property, the material is much more durable under constant stress. The tensile–deforming properties have so far been observed only in steel; therefore, zirconium dioxide can be colloquially called ceramic steel [6,13].

During preparation, the surface of zirconium oxide, before veneering ceramics are applied, is subjected to mechanical processing operations (machining–grinding, abrasive blasting) in order to increase the roughness and to improve the bond between the oxide substructure and the veneering ceramics. Thus, the main mechanism of bonding is surface machining and sandblasting [14]. After these processes, the surface is often subjected to chemical etching. The etching itself, as the chemical treatment of the surface, is less invasive, but not sufficient to obtain satisfactory results. For this reason, machining is required. Unfortunately, in spite of using stabilizers in the zirconium oxide structure during grinding and polishing, surface changes appear in its crystallographic structure (from tetragonal phase to a monoclinic phase), which adversely affect the properties of the material. During bonding, apart from mechanical attachments, physical adhesion may play a role. This is why both the values of surface free energy and the wettability after surface machining are important.

Mechanical surface treatment of zirconium oxide (for example, grinding and sandblasting) can cause an inflow of supercritical energy. This phenomenon causes surface distortions in the spatial lattice and, as a result, phase transformations into ZrO_2_. As a consequence, the complex stress, as well as subcritical crack growth, may build up on the flattened surface at the phase interface, thereby damaging or destroying the restoration [1]. Abrasive blasting is not neutral for a zirconium oxide surface. It leads to erosive destruction in the form of fissures, micro cracks, or ripping the ZrO_2_ grains out from the structure. The damaged area may have a range up to 10 μm in depth [19,20]. The status of the surface after sandblasting depends on parameters, such as the size and the type of grains, as well as the pressure and the angle of action of the blasting agent on the surface. Nevertheless, sandblasting is an important processing technique, which increases the roughness of the surface and it directly influences the connection quality [21]. Monoclinic ZrO_2_ has, in contrast to tetragonal ZrO_2_, a lower CET (coefficient of thermal expansion), amounting to approx. 7.5 × 10^−6^·K^−1^, which may also be important for the quality of the connection. Taking this fact into account, the connection between the framework and veneering ceramics is a shrink connection [1]. For the quality of the connection, substrate wettability and the related surface free energy (SFE) are important. To some extent, it shows the activity of the treated surface. Depending on the type of surface treatment, different values of this energy can be obtained. This should result in different bonding strengths between the framework and the veneering ceramics.

The human jaw handles large loads and artificial restorations may not always meet these high requirements. Over time, some ceramics become less resistant to stress and cracks form, even under regular loads [22]. The results of current clinical studies on the quality of the connection between zirconium oxide abutments and veneering porcelain indicate that there are common delaminations and chipping. The reasons for these phenomena are still not fully understood [21].

The consequences of the phase transition of zirconium oxide can be both positive and negative; it depends on the degree and the place of occurrence. To predict the direction of changes in zirconium oxide, the level of transformation during surface processing should be known. Mechanical processing, sandblasting, and grinding undoubtedly have adverse impacts. Due to the high chemical resistance of zirconium oxide, chemical treatment is not effective in terms of producing sufficient surface roughness, which is crucial for a good mechanical bonding between the framework and the ceramics. Sandblasting, in comparison to grinding, induces additional tension as a result of the percussion mechanism of action of abrasive particles. Thus, grinding should be favorable. Presently, there are no data about the transformations of zirconium oxide used in dental prosthetics depending on machining parameters. The objectives of this study were to evaluate the influence of grinding parameters on the degree of zirconium oxide transformation from the tetragonal to a monoclinic phase, and attempt to determine the depth of this transition. Surface free energy and its polar and dispersive components were also analyzed.

## 2. Materials and Methods

The material for the study consisted of 15 cylindrical samples of zirconium oxide 3Y-TZP Ceramill Zi (Amann Girrbach AG, Koblach, Austria). After being cut from a block, they were sintered in a furnace (Ceramill Therm; Amann Girrbach AG, Koblach, Austria) (8°/min from 200° to 1450°, 2 h at a constant temperature of 1450°). The whole sintering process lasted approx. 10 h. The material shrinkage totaled approximately 21%. After sintering, the diameter and the height of the samples were 20 and 10 mm, respectively. The compositions of the samples are shown in Table 1.

After the sintering process, the samples were divided into 5 groups and, within each group, the surfaces of the samples were subjected to the following treatments:

A—coarse grinding—dry grinding wheel with grit 120,

B—coarse grinding—wet grinding wheel with grit 120,

C—fine grinding—dry grinding wheel with grit 500,

D—fine grinding—wet grinding wheel of grit 500,

E—polishing.

An initial sample was used as a reference sample. After milling, the sample was referred to as F. The samples prepared in this way were subjected to the following tests:

- qualitative and quantitative diffractometric tests determining the phases occurring in individual samples and calculating their content,
- measurements of the contact angle and free energy of the surface.

Diffraction tests were performed on a PANalytical Empyrean X-ray diffractometer (Malvern Panalytical Netherlands, Lelyweg 1, EA Almelo, Netherlands). The diameter of the goniometer was 240 mm, the device worked in the Bragg–Brentano geometry in the θ-θ system (hereinafter referred to as S) or in the geometry of a constant angle of incidence (hereinafter referred to as GI). The primary beam was obtained using an X-ray tube with a cobalt (Co) anode emitting characteristic radiation with a wavelength of λ = 1.79 Å. To obtain a parallel beam, a Goebel mirror was used. The remaining elements of the primary beam optics were a 1/2 degree divergence slit, a 1.4 mm anti scatter slit, a 0.04 rad Soller slit, and a 10 mm mask. The intensity of the scattered beam was recorded with a proportional Xe detector equipped with a PPC collimator and a Soller slit of 0.04 rad. The samples were placed on an X-Y-Z-Phi-Chi five-axis universal stage enabling precise alignment of the specimens by adjusting their height and tilt angle, which depended on the plane parallelism of the tested surfaces. Tests were carried out in the angular range of 2θ = (25°–95°) with a step of 0.05° and a 2-s time step. Measurements in the geometry of the constant angle of incidence were carried out under exactly the same conditions, with the angle of incidence of the primary beam being 5° in the whole range of 2θ. The qualitative and quantitative phase analysis of the obtained diffractograms were performed using the High Score Plus software, supplied by the diffractometer manufacturer, and the ICDD PDF4 + crystallographic database.

The surfaces of all samples were subjected to SEM-BEIW observations with use of a JEOL JSM-6610LV scanning electron microscope (JEOL, Tokyo, Japan). The examined surfaces were imaged under an accelerating voltage of 20 kV. Microanalyses of the chemical compositions were performed with an EDS X- MAX 80 micro analyzer (Oxford Instruments, Oxford, UK) under an accelerating voltage of 20 kV. According to the EDS method, we performed a quantitative analysis of the chemical composition of the selected areas.

The determination of the surface free energy was carried out by depositing drops of water (polar liquid) and diiodomethane (apolar liquid) with a volume of 3 μL. Pictures of the drops on individual samples were taken, which allowed the determination of the wetting angle needed to calculate the free energy of the surface. The SFE calculations were made by dividing into the dispersion (apolar) component, the polar component, and the total surface energy. The Owens–Wendt model was used to calculate the values of individual dispersion and the polar components of the tested samples [23,24].

## 3. Results

**Figure 1:**
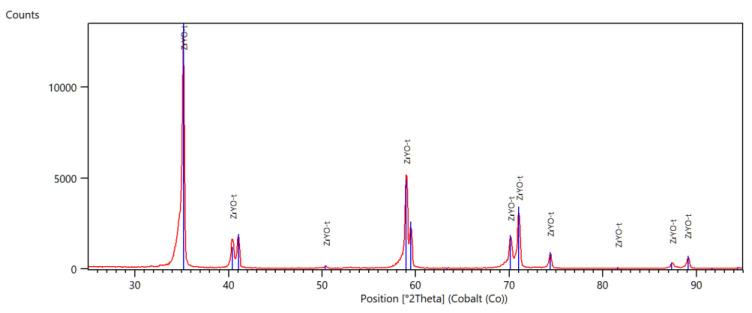
Diffractogram of a milled sample, F.

**Figure 2:**
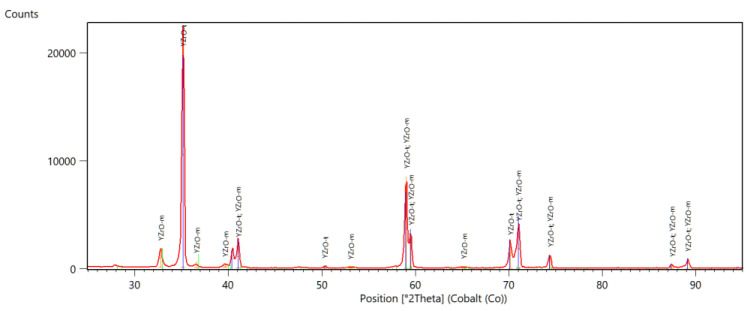
Diffractogram of a ground sample, B.

**Figure 3:**
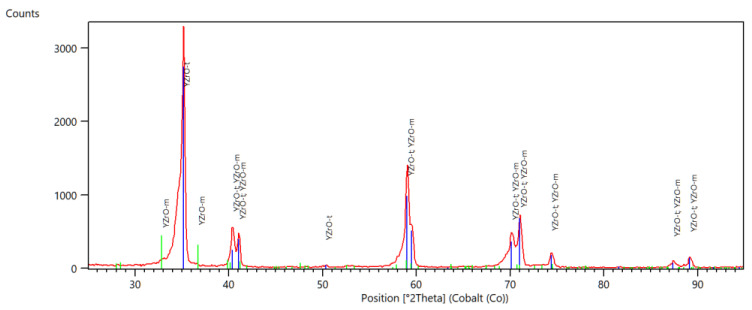
Diffractogram of a polished sample, E.

In Figure 1, Figure 2 and Figure 3, selected diffractograms are shown, from which the content of the monoclinic phase in the tested samples was calculated.

The presented diffractograms show that, in the comparative (milled) sample F, only reflections from the tetragonal phase are present. In the remaining samples, apart from reflections of the tetragonal phase, there are also reflections of the monoclinic phase. The intensity of these reflections varies, which is due to the different contents of the individual phases. Table 2 shows the amount of monoclinic phase in the tested samples

Table 3 presents the measured contact angles of individual samples, while Table 4 shows the calculated results of the surface free energy.

In all the tested samples, it was observed that the diiodomethane contact angles (apolar liquid) were smaller than the water contact angles (polar liquid). Moreover, the size of the polar component was smaller than that of the apolar component.

**Figure 4:**
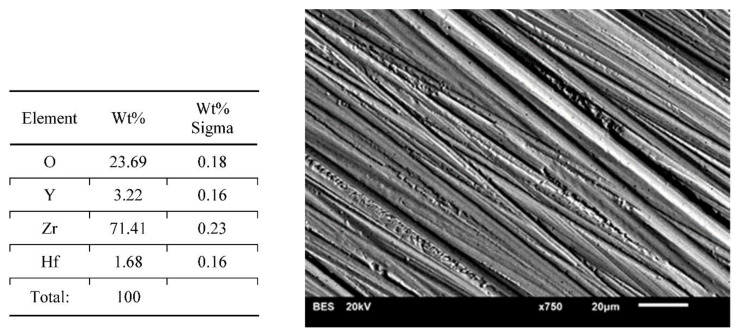
Chemical composition in presented area and a backscattered electrons image of sample—dry ground 120 grit paper. Coarse features and small arrachments on a surface.

**Figure 5:**
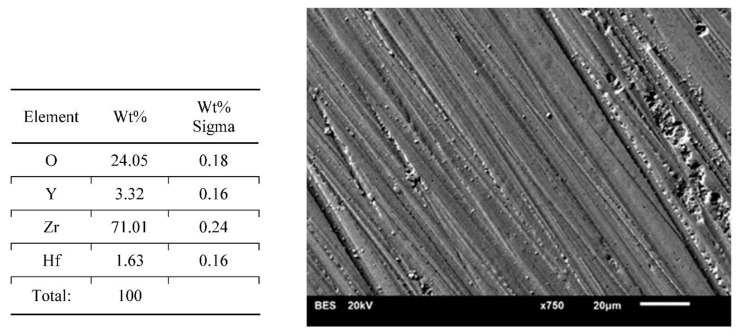
Chemical composition of the presented area and a backscattered electrons image of a sample—wet ground 120 grit paper. Wide features and big arrachments visible.

**Figure 6:**
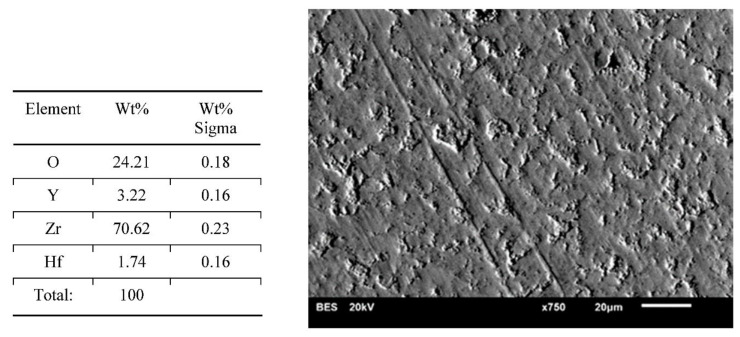
Chemical composition of the presented area and a backscattered electron image of a sample—dry ground 500 grit paper. Small features following lots of fine arrachments of the material.

**Figure 7:**
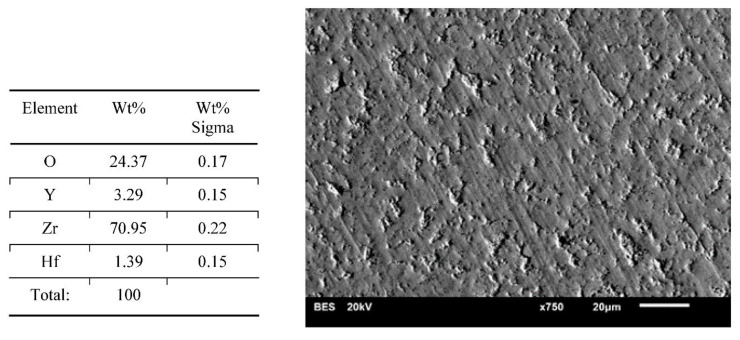
Chemical composition of the presented area and a backscattered electron image of a sample—wet ground 500 grit paper. Lots of small splintering of the material with small and less visible features.

**Figure 8:**
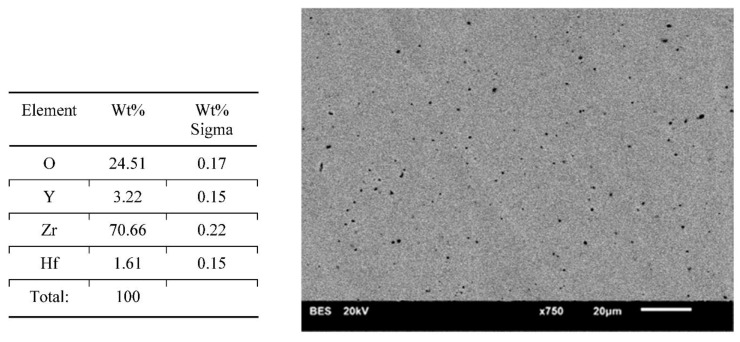
Chemical composition of the presented area and a backscattered electron image of the polished sample. A smooth surface of the sample with the tiny cavities in the material which can correspond to arrachments or the porosity of the material. No features observed.

Results of surface characterization by scanning electron microscope are presented in Figure 4, Figure 5, Figure 6, Figure 7 and Figure 8. The chemical compositions in the studied areas and backscattered electrons images are shown.

Chemical composition values of the ground samples did not reveal any significant changes, regardless of the method of surface preparation. Obtained values were typical for such types of ceramics, namely YSZ (yttria stabilized zirconia).

## 4. Discussion

The debate on the status of the dental materials surface as well as the studies on its quality are of a great importance. The evaluation of the material properties and their durability are clinically relevant [23].

Diffractometric studies have shown that the mechanical treatment of zirconium oxide causes transformation of the tetragonal phase into monoclinic phase on its surface. The content of the monoclinic phase in the samples subjected to grinding is similar, while in the polished samples it is lower. There were no significant differences in the amount of monoclinic phase in samples treated with discs of different grain size and samples ground with/without water cooling. As expected, the starting sample did not contain the monoclinic phase. Differences in the amount of monoclinic phase, calculated from the diffractogram, depending on the sample scanning method were also observed. In all the tested samples, the amounts of the phase calculated from the diffractogram at the constant angle of incidence are slightly higher than those calculated from the Bragg-Brentano geometry scans.

All samples were tested in symmetrical diffraction geometry and grazing incidence geometry. The significant differences in calculated amounts of monoclinic phases (by Rietveld method) can be observed dependent on X-ray beam penetration depth.

**Figure 9:**
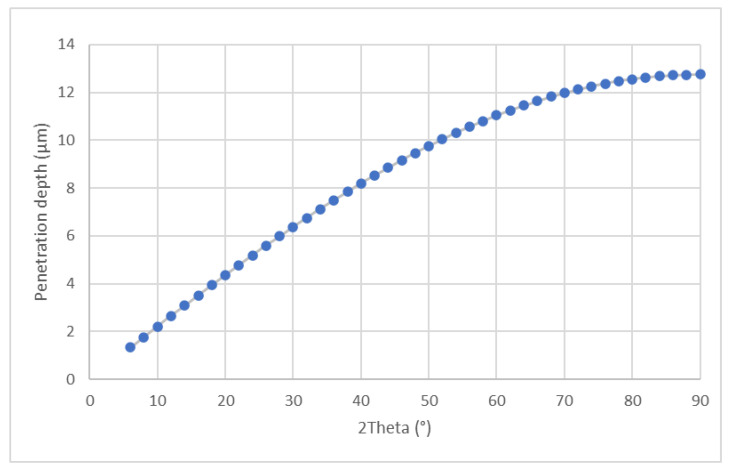
Penetration depth plot for symmetrical diffraction.

**Figure 10:**
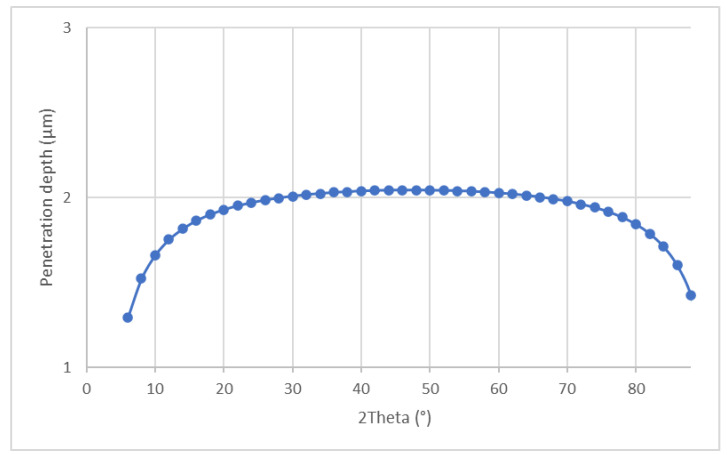
Penetration depth plot for grazing incidence diffraction-incident angle 5°.

The plots of penetration depths are presented in Figure 9 and Figure 10, for symmetrical and constant incidence angle, respectively.

In case of symmetrical scan the penetration depth was calculated using the formula:

$$PD=\frac{ln(\frac{1}{1-G_{x}})}{2\mu }sin\theta$$

where:

*G_x_*—the intensity diffracted by the layer considered as a fraction of the total integrated intensity diffracted by a specimen of infinite thickness

*μ*—linear absorption coefficient

*θ*—Bragg angle

For constant angle of incidence:

$$PD=\frac{-\ln(\frac{1}{1-G_{x}})}{\mu \left[\frac{1}{sin\alpha }+\frac{1}{sin\left(2\theta -\alpha \right)}\right]}sin\theta$$

where *α*—is the angle of incidence—(used in measurements—5°).

In both cases *G_x_* was taken 0.95, *μ* = 1175 [1/cm].

The differences in calculated amounts of monoclinic phase are caused by a fact that symmetrical diffraction gives an average result from the whole illuminated volume of material, while grazing incidence method allows determining the amount of phases at particular depth. Comparison of results indicated the presence of concentration gradient of a monoclinic phase towards the core of samples.

The results of the study indicate that the transition from the tetragonal phase into a monoclinic one, which is a consequence of surface machining, occurs at a certain depth depending on the type of processing. The bigger the grains used for processing, the greater the depth of the transformation. This leads to the different mechanical properties of the material. No significant influence of cooling on the degree and the depth of the transition was observed.

Summarizing the results of the diffraction tests based on zirconium oxide substructure–dental veneering ceramics, it should be stated that the mechanical treatment performed, in order to develop the surface and increase roughness, resulted in the appearance of an unfavorable monoclinic phase in the surface layers, which may have an adverse effect on the quality of the connections. It was confirmed by Guzzato et al. [25] in their studies regarding the influence of the surface and heat treatment of the material. They demonstrated via diffractometric analyses that sandblasting influences the transformation of tetragonal phase into a monoclinic one to a greater extent than the grinding. However, this transition occurs in both processes.

There are studies in which, after sandblasting, the grains of the abrasive can be stuck in the surface of the zirconium oxide. Even though the number of embedded grains is less than in case of metal machining, it nevertheless may result with a worsening of the adhesion [26]. Despite the many advantages of zirconium oxide, there are more failures in terms of adhesion to the veneering material in comparison with restorations using traditional metal substructures. According to the literature, the most common drawbacks are cracks, chipping, and fractures in the ceramics. The least likely to occur are delimitations of the material. Small chips require a slight grinding and polishing, but bigger defects result in needing to exchange the whole restoration [27,28].

Scanning electron microscope images of the evaluated surfaces demonstrate that the changes in its topography depend on the type of machining. The bigger the grain is, the more developed the surface of the sample is. This undoubtedly impacts the connection quality with veneering ceramics. From this point of view, grinding with discs with a coarse grain is most beneficial. The occurring deeper fissures create better mechanical attachments than flat scratches after using small-grit discs. On the surfaces of the polished and ground samples with small grains, small local defects in the material occurred. The most likely is the effect of ripping molecules out of the material substrate. These kinds of defects are also seen on the surfaces of samples prepared with coarse grains; however, they are fewer and occur occasionally. Even though cooling had no significant influence on the transition of the tetragonal phase into the monoclinic phase, it affected the surface appearance. In samples prepared with water cooling there was less damage in comparison with the items with no cooling treatment. These defects may be beneficial as they increase the development of the surface, improving mechanical bonding with the veneering ceramics. There are no data whether an unfavorable transition of phases is happening in their neighborhood. Unfortunately, on the basis of the cumulative image (not the local one) of XRD examination, we cannot determine this. Perhaps electron backscatter diffraction could be more accurate and useful. The analysis of the chemical compositions of the prepared surfaces did not reveal significant changes. The compositions of all samples were within the limits set out by the specifications of the material manufacturer.

In prosthetics, the important factors in determining the quality of a connection is wettability and free surface energy. Two aspects of wettability were considered: wettability with water—due to the fact that porcelain is applied in ambient temperatures in the form of a suspension (the polar element of SFE matters) and wettability with melted ceramics during the sintering process (apolar element is important). The proper wettability values provide adequate penetration of the material into the pores of zirconium oxide created by processing and an appropriate distribution on the surface [29,30].

The measurements of the contact angle and free energy of the surface did not show any significant differences between the individual samples. The contact angles of the samples ranged from 67° to 79° for water (polar liquid) and from 47° to 61° for diiodomethane (apolar liquid). In all the tested cases, it can be said that both liquids wetted the surfaces of the tested samples (θ < 90°). In all the treated samples, better wettability was observed with the non-polar liquid as opposed to the polar liquid. The calculated values of the surface free energies were similar for all samples and fell within the ranges of 8–11 mJ/m^2^ for the polar component, 27–36 mJ/m^2^ for the apolar component, and 37–43 mJ/m^2^ for the surface free energy. In all cases, the value of the polar component is lower than the value of the apolar component. The values of the individual components of the surface free energy should be taken into account when designing the composition of the facing ceramics. The higher value of the apolar component indicates an affinity for apolar materials. A greater value of the apolar component relative to the polar one means that during an application of the ceramics at room temperature, it does not flow into surface irregularities. However, the flow of the material will improve during firing, which makes a proper connection.

Obtained results indicate that the type of machining and its parameters directly influence the phase transformation in a zirconium oxide structure. Therefore, it determines the success of the material–veneering ceramic connection.

## 5. Conclusions

(1) Mechanical processing affects the surface condition and induces changes in the crystallographic structure of zirconium oxide samples. It causes an unfavorable transformation of the tetragonal phase into a monoclinic phase in the surface layers, which may deteriorate the bonding quality of veneering ceramics.
(2) The value of the surface free energy after surface treatment shows no significant differences.
(3) The higher value of the surface free energy dispersion component proves that an eligible connection of zirconium oxide with ceramics has apolar properties.
(4) Flowing ceramics, being an apolar liquid in firing temperatures, will guarantee a good long-term connection with ZrO_2_.

## Figures and Tables

**Table 1:** Composition of the tested material (according to the manufacturer’s information).

ZrO_2_ + HfO_2_ + Y_2_O_3_	>99.9
Y_2_O_3_	4.5–5.4
HfO_2_	<5
Al_2_O_3_	<0.5
other oxides	<0.5

**Table 2:** Content of the monoclinic phase in the tested samples.

Sample	Scan Type	Monoclinic Phase Content [%]
A	S	9
	GI	12
B	S	10
	GI	12
C	S	13
	GI	15
D	S	13
	GI	16
E	S	5
	GI	7
F	G	0
	SKP	0

**Table 3:** Contact angles of sample surfaces with water and diiodomethane.

Sample	Θ_w_ (deg)	Θ_j_ (deg)
A	69.80 ± 1.03	54.14 ± 0.48
B	70.15 ± 1.15	54.23 ± 1.21
C	73.39 ± 2.14	61.09 ± 1.56
D	73.62 ± 2.35	56.52 ± 0.86
E	76.81 ± 2.33	57.49 ± 2.21
F	75.51 ± 3.34	47.53 ± 3.46

**Table 4:** Values of free energy of surfaces.

Sample	Polar Component (mJ/m^2^)	Dispersion Component (mJ/m^2^)	Surface Free Energy (mJ/m^2^)
A	10.41 ± 1.87	31.78 ± 0.41	42.39 ± 1.71
B	9.54 ± 0.86	31.47 ± 1.47	41.18 ± 0.97
C	8.26 ± 2.13	32.17 ± 0.56	40.26 ± 1.63
D	10,21 ± 3.23	27.26 ± 1.27	37.12 ± 3.11
E	7.43 ± 1.31	30.41 ± 1.75	37.69 ± 1.78
F	7.29 ± 2.65	35.54 ± 3.12	42.81 ± 2.23

## Data Availability

The data presented in this study are available on request from the corresponding author.
